# Chimeric antigen receptor T-cell therapy in glioblastoma: charging the T cells to fight

**DOI:** 10.1186/s12967-020-02598-0

**Published:** 2020-11-11

**Authors:** Craig A. Land, Phillip R. Musich, Dalia Haydar, Giedre Krenciute, Qian Xie

**Affiliations:** 1 Department of Biomedical Sciences, Quillen College of Medicine, East Tennessee State University, Johnson City, TN 37614 USA; 2 Department of Bone Marrow Transplantation and Cellular Therapy, St. Jude Children’s Research Hospital, Memphis, TN 38105 USA; 3 Center of Excellence for Inflammation, Infectious Disease and Immunity, Quillen College of Medicine, East Tennessee State University, Johnson City, TN 37614 USA

**Keywords:** Chimeric antigen receptors, CAR, T-cell therapy, Glioblastoma, Cellular immunotherapy

## Abstract

Glioblastoma multiforme (GBM) is the most common malignant brain cancer that invades normal brain tissue and impedes surgical eradication, resulting in early local recurrence and high mortality. In addition, most therapeutic agents lack permeability across the blood brain barrier (BBB), further reducing the efficacy of chemotherapy. Thus, effective treatment against GBM requires tumor specific targets and efficient intracranial drug delivery. With the most recent advances in immunotherapy, genetically engineered T cells with chimeric antigen receptors (CARs) are becoming a promising approach for treating cancer. By transducing T lymphocytes with CAR constructs containing a tumor-associated antigen (TAA) recognition domain linked to the constant regions of a signaling T cell receptor, CAR T cells may recognize a predefined TAA with high specificity in a non-MHC restricted manner, and is independent of antigen processing. Active T cells can travel across the BBB, providing additional advantage for drug delivery and tumor targeting. Here we review the CAR design and technical innovations, the major targets that are in pre-clinical and clinical development with a focus on GBM, and multiple strategies developed to improve CAR T cell efficacy.

## Background

Glioblastoma (GBM), WHO grade IV glioma, is the most devastating brain tumor in adults [1]. The intrinsic capability of single tumor cells to invade normal brain tissue impedes surgical eradication, predictably resulting in early local recurrence and death. Current treatment options for GBM include maximal surgical removal, radiotherapy and chemotherapy. However, the overall survival rate has not changed significantly over the past decade.

Adoptive cell transfer (ACT) using autologous lymphocytes for treating cancer started in 1988 with metastatic melanoma patients [2]. While early trials reported an overall response in approximately 34% of patients [3], subsequent studies demonstrated that lymphodepletion prior to ACT may significantly improve the response rates to about 50% of patients [4, 5]. During the same period, genetically engineered T cell receptors (TCR) were explored to achieve robust T cell responses which ultimately led to the emergence of chimeric antigen receptor (CAR) T-cell therapy [6, 7]. In 2017, CD19-CAR (tisagenlecleucel) became the first FDA-approved CAR-T cell therapy for treating patients with relapsed or refractory B cell acute lymphoblastic leukemia (ALL) [8].

The first ACT-based attempts for GBM therapy started about 40 years ago when several case reports showed that autologous leukocyte infusion into the resection cavity at the time of tumor resection may improve patient’s survival without toxicity [9–11]. However, these approaches lacked specificity. To overcome this, genetic engineered T cells have been generated to specifically target brain tumor associated antigens (TAA) and have shown promising pre-clinical results. For GBM, interleukin-13 receptor alpha 2 (IL13Rα2) became the first CAR T-cell target tested in the clinic for its specific overexpression in tumors but not in normal tissues [12]. Epidermal growth factor receptor variant III (EGFR*vIII*) [13], human epidermal growth factor 2 (HER2) [14] and erythropoietin-producing hepatocellular carcinoma A2 (EphA2) [15] were also developed as major targets for their overexpression in GBM but not in healthy brain tissues. Other newly developed targets include ganglioside 2 (GD2) [16, 17], B7-H3 [18] and chlorotoxin [19]. Although all these targets have shown promising preclinical results and are evaluated in clinical trials (see Table 1 for summary), lack of clinical efficacy indicates that genetic modifications and therapeutic combinations are required in order to achieve a better response. In this review, we will introduce the basic CAR structures and their optimizations, the major targets that are in pre-clinical and clinical development for targeting GBM, and the combination strategies to improve CAR T-cell efficacy.

**Table 1:** Recent clinical trials of CAR T-cell therapy in glioblastoma (in alphabetical order)

Target	Study title	Phase	Status	Institution and location
CAR T-cell therapy				
B7-H3	Pilot study of B7-H3 CAR-T in treating patients with recurrent and refractory glioblastoma. NCT04385173	1	Recruiting	Second Affiliated Hospital, School of Medicine, Zhejiang University, China
	B7-H3 CAR-T for recurrent or refractory glioblastoma. NCT04077866	1/2	Recruiting	Second Affiliated Hospital, School of Medicine, Zhejiang University, China
Chlorotoxin	Chimeric antigen receptor (CAR) T cells with a cholrotoxin tumor-targeting domain for the treatment of MPP2^+^ recurrent or progressive glioblastoma. NCT04214392	1	Recruiting	City of Hope Medical Center, CA, United States
EGFRvIII	CAR T cell receptor immunotherapy targeting EGFRvIII for patients with malignant gliomas expressing EGFRvIII. NCT01454596	1/2	Completed	National Institutes of Health Clinical Center, United States
	Intracerebral EGFR-vIII CAR-T cells for recurrent GBM. NCT03283631	1	Suspended	Duke University Medical Center, NC, United States
	Pilot study of autologous anti-EGFRvIII CAR T cells in recurrent glioblastoma multiforme. NCT02844062	1	Unknown status	Sanbo Brain Hospital Capital Medical University, Beijing, China
	Memory-enriched T cells in treating patients with recurrent or refractory grade III–IV glioma. NCT03389230	1	Recruiting	City of Hope Medical Center, CA, United States
EphA2	CAR-T cell immunotherapy for EphA2 positive malignant glioma patients. NCT02575261	1 / 2	Withdrawn	Central Laboratory in Fuda Cancer Hospital, Guangdong, China
GD2	C7R-GD2.CAR T cells for patients with GD2-expressing brain tumors (GAIL-B). NCT04099797	1	Recruiting	Baylor College of Medicine, TX, United States
	Personalized chimeric antigen receptor T cell immunotherapy for patients with recurrent malignant gliomas. NCT03423992	1	Recruiting	Xuanwu Hospital, Beijing, China
HER2	Intracranial injection of NK-92/5.28.z cells in patients with recurrent HER2-positive glioblastoma. NCT03383978	1	Recruiting	Senckenberg Institute of Neurooncology, Frankfurt, Germany
	CMV-specific cytotoxic T lymphocytes expressing CAR targeting HER2 in patients with GBM. NCT01109095	1	Completed	Baylor College of Medicine, TX, United States
IL13Ra2	Genetically modified T cells in treating patients with recurrent or refractory malignant glioma. NCT02208362	1	Recruiting	City of Hope Medical Center, CA, United States
	Phase I study of cellular immunotherapy for recurrent/refractory malignant glioma using intratumoral infusions of GRm13Z40-2, an allogeneic CD8^+^ cytolitic T-cell line genetically modified to express the IL 13-Zetakine and HyTK and to be resistant to glucocorticoids, in combination with interleukin-2. NCT01082926	1	Completed	City of Hope Medical Center, , CA, United States
CAR T-cell therapy in combination with chemotherapy				
EGFRvIII	Immunogene-modified T (IgT) cells against glioblastoma multiforme. NCT03170141	1	Enrolling by invitation	Shenzhen Geno-immune Medical Institute, Guangdong, China
CAR T-cell therapy in combination with immune check point inhibitors				
EGFRvIII	CART-EGFRvIII + pembrolizumab in GBM. NCT03726515	1	Active, not recruiting	Abramson Cancer Center of the University of Pennsylvania, PA, United States
IL13Ra2	IL13Ralpha2-targeted chimeric antigen receptor (CAR) T cells with or without nivolumab and ipilimumab in treating patients with recurrent or refractory glioblastoma. NCT04003649	1	Recruiting	City of Hope Medical Center, CA, United States

Most recent GBM CAR-T cell therapy clinical trials were searched at www.Clinicaltrials.gov (2010 to present)

## CAR designs and optimization

### Conventional CARs

**Fig. 1:**
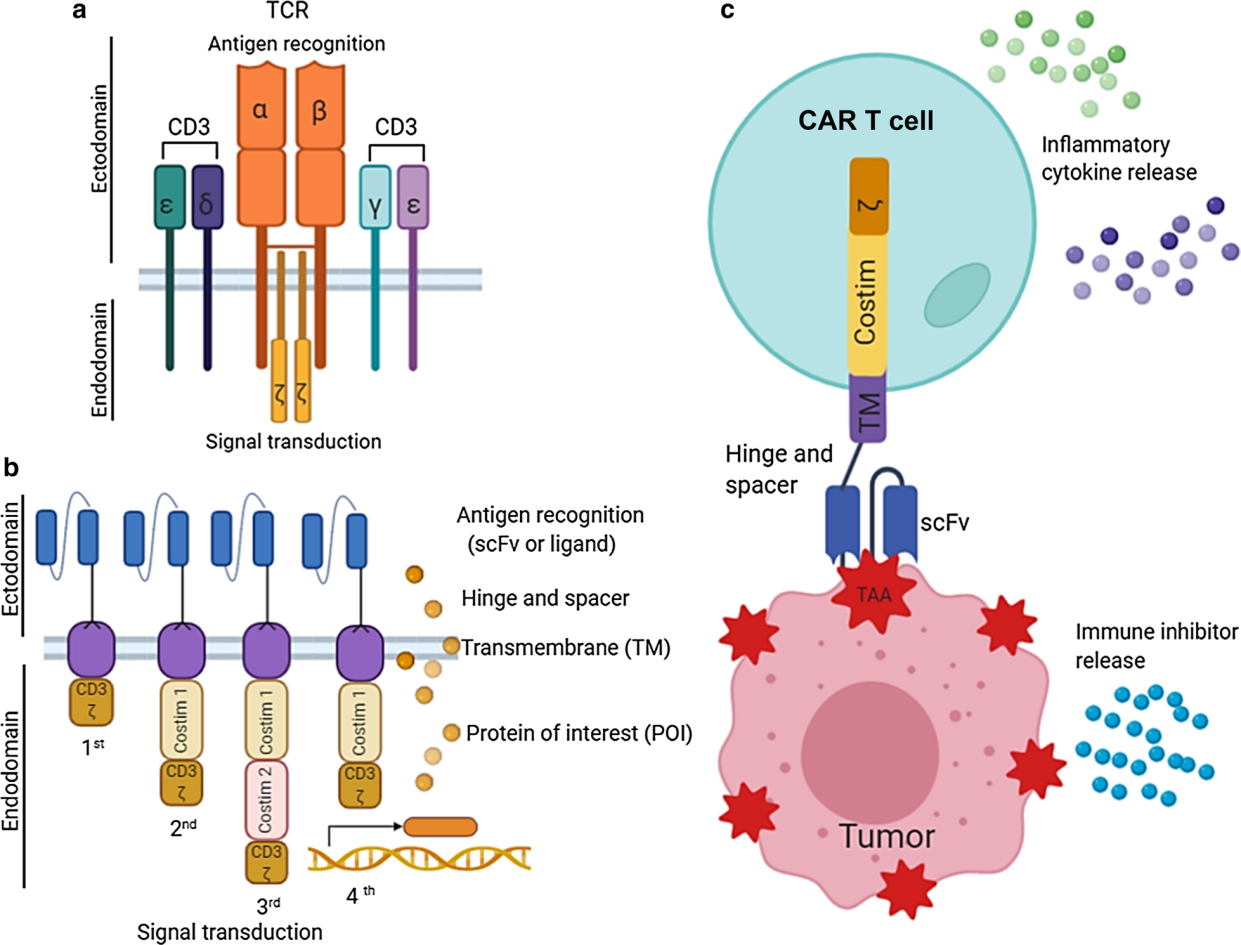
Basic principle of CAR structure and CAR T-cell therapy. **a** T-cell receptor (TCR) complex. The TCR-CD3 complex contains variable TCR-α and TCR-β chains coupled to three dimeric signaling transduction modules CD3 δ/ε, CD3 γ/ε and CD3 ζ/ζ. T cell activation starts when the TCR binds to the antigen/MHC peptide on the surface of antigen-presenting cells or tumor cells. **b** Basic 1st–4th generation design of chimeric-antigen receptors (CAR). The ectodomain of the CAR is composed of an antigen-binding region (scFv), a hinge and a spacer. The transmembrane portion links the ectodomain to the intracellular endodomain. The endodomain contains a CD3ζ signaling domain (1st generation) and one or two costimulatory domains (2nd and 3rd generation, respectively). The 4th generation CARs (also called TRUCKs) contain an additional expression vector or a transgene to express a synthetic protein of interest (POI), for instance cytokines and chemokines. **c** Mechanism of CAR T-cell therapy. CAR T cells use the scFv domain of the CAR to recognize and bind to the tumor-associated antigen (TAA) on the tumor cell surface. This binding activates CAR T cells signaling through the endodomain CD3ζ module which elicits cytotoxic functions by producing perforins, granzymes and cytokines

T cells play an essential role in cell-mediated immune responses. T cell activation requires two signals: (i) TCRs recognize and bind to the antigen peptides presented on the major histocompatibility complex (MHC) exposed on the surface of antigen-presenting cells or tumor cells, and (ii) T cell co-stimulatory signaling modules, such as CD28, 4-1BB and OX40, which bind to the ligands expressed on antigen-presenting cells. It is well accepted that T cells play a crucial role in immune surveillance, a function to detect and eliminate tumor cells from the host. Compared to the TCR (Fig. 1a), a CAR is a synthetic molecule designed to express an antigen recognition domain which is usually a single-chain variable fragment (scFv) or a ligand specific for one TAA [20, 21]. This ectodomain is linked via a hinge and spacer to the transmembrane domain and an intracellular signaling domain. The endodomain of the CAR consists of CD3ζ activation domain (1st generation CAR) and one or two co-stimulatory domain such as CD28, 4-1BB, or OX40 (2nd or 3rd generation CAR). While first generation CAR T cells had limited longevity and tumor killing efficacy in vivo [6], the second and third generation CAR T cells showed improved proliferation and effector function [22]. The 4th generation of CARs further compose additional genetic modifications that allow the release of transgenic proteins of interest (POI), such as cytokines, to enhance the CAR T cell expansion and survival [23] (Fig. 1b). Most importantly, with this design, CAR T cell can be re-directed to target specific tumor cells via T cell-mediated multi-functional killing activities independent of MHC expression levels (Fig. 1c). Loss of MHC class I expression in tumor cells is a common mechanism leading to tumor escape and resistance to T cell immunity [24]. The fact that CAR T cells can recognize and bind to the TAAs unrestricted to MHC class I expression is making the CAR T-cell therapy an attractive approach for anti-cancer therapeutics.

### Universal CARs

**Fig. 2:**
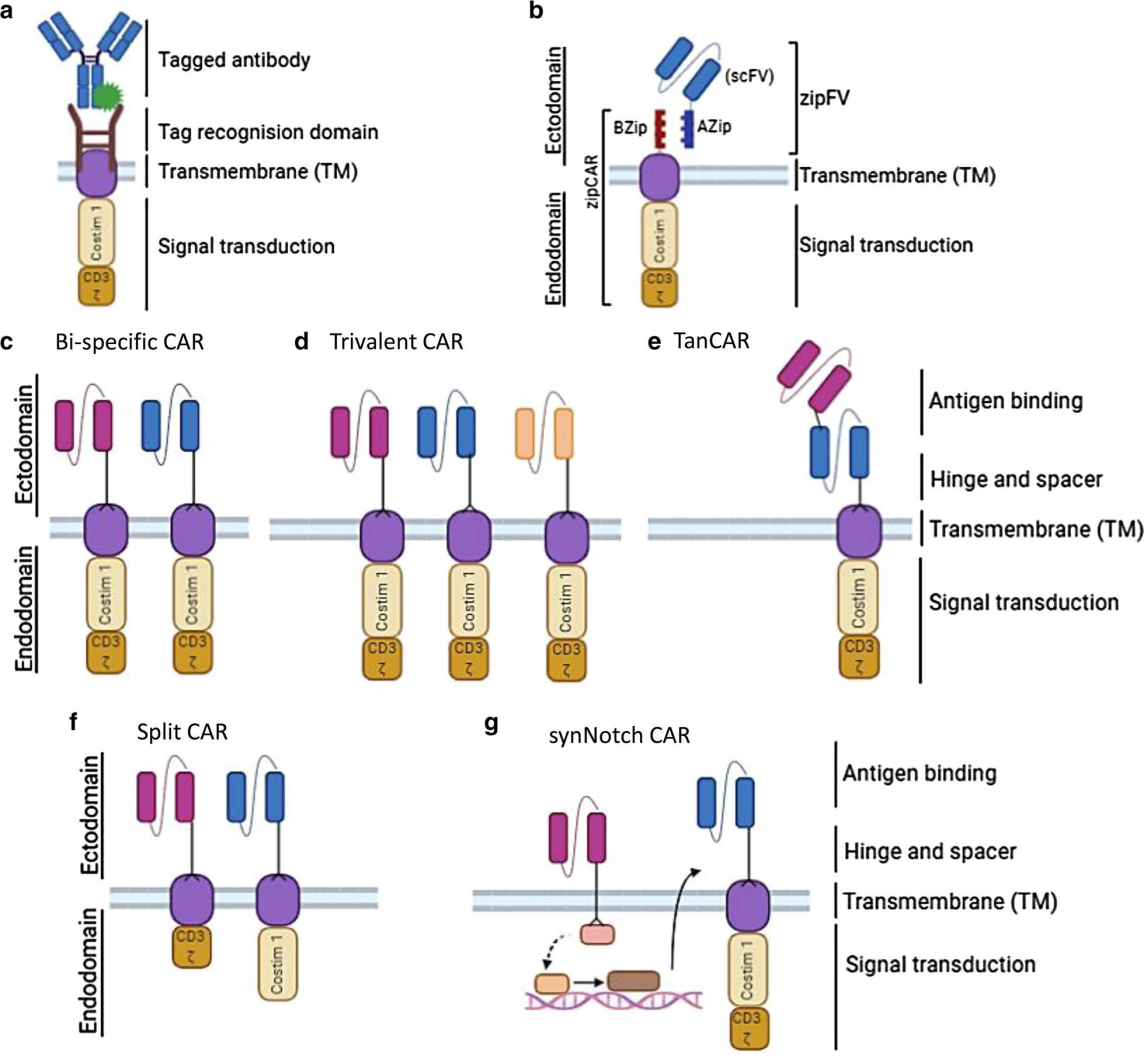
Optimized CAR designs. **a** The anti-tag uCAR is designed to express an antigen recognition domain specific for a tag (often FITC) molecule attached to a monoclonal antibody specific to the TAA on the surface of tumor cells. **b** SUPRA CAR is composed of two parts: the zipFv component consists of a scFv specific for the TAA to be targeted and a leucine zipper (AZip). The second component is zipCAR-T cell expressing a CAR with an extracellular leucine zipper (BZip). The zipFv binds to the TAA via the scFv domain and to the zipCAR via binding of the AZip and BZip leucine zipper domains, leading to CAR T cell activation. **c**, **d** Bi-specific or trivalent CAR T cells are designed to co-express two or three CARs within the same cell that are directed to two or three different brain TAAs, respectively. Alternatively, two or three different CAR T cell populations could be pooled together to simultaneously target multiple TAAs on the tumor cell surface. **e** Tandem CAR is composed of two or more scFvs in tandem followed by hinge, transmembrane and signaling domains. Binding of either one or more TAAs may fully activate T cell signaling and function. **f** Split CAR is designed to co-express two different CARs for targeting two different TAAs. One CAR contains the CD3ζ signal and the other contains the co-stimulation signal. Binding to both TAAs is required for CAR T cell activation. **g** The activation of a synNotch CAR T cell requires two TAAs be present on the cancer cell surface and occurs in two steps: (1) the synNotch receptor recognizes and binds to the first antigen, leading to release of a transcription activator for the CAR transcription; and (2) the CAR recognizes and binds to the second antigen, leading to full CAR T cell activation. Only when both antigens are present will the T cells be activated and kill the target tumor cells

CAR T cells are showing promising anti-tumor activity against solid and brain tumors, however, the generation of a CAR targeting a novel tumor antigen is complicated and time consuming. It is well established that every new antigen-recognition domain has to be tested with multiple co-stimulatory domains. Even if well-defined and characterized, signaling domains might not be fully functional when testing with a new antigen-recognition domain. In addition, multiple studies show that the hinge and transmembrane domains also influence the function of an antigen-specific CAR T cell [25–27]. One way to overcome these challenges is to create a universal CAR (uCAR). Indeed, several groups have reported generation of uCARs. By engineering the CAR vector to express an antigen-recognition domain specific for fluorescein isothiocyanate (FITC), Tamada et al*.* [28] has reported an anti-FITC uCAR which further binds to FITC-tagged monoclonal antibody specific to HER2, or CD20 (Fig. 2a). Most importantly, the activity of the anti-FITC uCAR can be attenuated by additional injection of FITC-IgG, thus providing a safety switch when the risk of toxicity increases. Their study demonstrates that anti-tag CAR may elicit a potent anti-tumor activity in vitro and in vivo*.* However, it is not clear if such a design affects CAR T cell effector function when compared to a conventional CAR design.

Another uCAR study by Cho et al*.* [29] reported a split, universal and programmable (SUPRA) CAR system (Fig. 2b). This is a two-component system: one component is a zipFv that contains a TAA-specific scFv that is linked to a leucine zipper (AZip); the other component is a CAR T cell that contains an extracellular leucine zipper (BZip) plus a transmembrane domain and an intracellular signaling domain (zipCAR) (Fig. 2b). After the scFv domain of the zipFv recognizes and binds to the specific TAA on tumor cells, the BZip on the zipCAR T cells can bind the AZip leucine zipper of the zipFv, leading to a fully functional CAR T-cell activation and signaling. Similar to the anti-FITC uCAR, the activity of zipCAR can be attenuated by subsequent injection of the AZip peptide. With this design, the authors generated a SUPRA CAR specific for HER2 which showed potent tumor killing activity in solid tumor settings. Moreover, they further modified this system to target multiple antigens.

While both groups demonstrated their uCAR systems for targeting HER2 experimentally, brain tumor-specific studies are warranted to evaluate whether uCARs are feasible for clinical use, given a very complex and hostile brain tumor microenvironment. In addition, the biological activity of a uCAR requires a stable complex formed by three components instead of two in traditional CARs, raising the concerns of stability and functional activities when tested in vivo. Finally, future studies should evaluate carefully the safety of uCARs as both systems contain immunogenic molecules such as FITC and leucine zippers.

### CARs targeting multiple antigens

Despite the increasing number of targets being tested in clinical trials (Table 1), CAR T cells have failed to completely eradicate brain tumors. A major limitation of a single-antigen targeting CAR T-cell therapy for GBM is the inherent heterogeneity and plasticity of the tumor cells, allowing some cells to escape CAR T cell killing due to the loss of the targeted antigen. As a result, single antigen-targeting CAR T cells fail to completely eradicate brain tumors resulting in antigen-negative relapses as demonstrated by pre-clinical and clinical studies [30]. Such relapses prompted investigators to devise strategies and design CARs that can target multiple TAAs, including bi-specific, trivalent, tandem, split, and synNotch CARs (Fig. 2c–g). Among them, the first three strategies have been explored for targeting brain tumors.

Bi-specific CAR T cells are generated by either double transduction of two CARs that are directed against two different brain TAA (co-expression) or by pooling two different CAR T cell populations (pooled CAR T cells) (Fig. 2c). Hedge et al*.* [31] generated T cells by (i) individually expressing HER2- and IL13Ra2-specific CARs or (ii) co-expressing both CARs, one targeting each antigen. They revealed that both approaches elicit an adequate anti-tumor response and, most importantly, prevented antigen escape in GBM in vitro and in vivo. Moreover, T cells co-expressing HER2- and IL13Ra2-CARs showed better anti-tumor response when compared to T cells individually expressing both CARs. The same group took it a step further and designed a trivalent CAR structure which encodes three full CAR molecules in one construct (Fig. 2d). Thus, a single transduction allowed them to express three different, separate CARs simultaneously in one T cell. In their study, using this method, Bielamowicz et al*.* [32] reported a trivalent CAR T-cell system for co-targeting HER2, IL13Ra2, and EphA2; the trivalent CAR T-cell therapy lead to a significantly better overall survival in vivo when compared to dual and single CAR T cells when tested in patient derived xenograft models.

Another multi-antigen targeting approach, also established by the same group, is the tandem CAR (TanCAR). Here, the CAR is composed of multiple scFvs placed in tandem followed by hinge, transmembrane and signaling domains (Fig. 2e). Hedge et al. [33] reported the development of a TanCAR designed to target HER2 and IL13Rα2 against GBM. The authors demonstrated that TanCAR T cells have a synergistic effect in eliminating glioma cells when compared to CAR T cells individually targeting either IL13Rα2 or HER2 antigens. In addition, simultaneous binding to both antigens in the TanCAR system has shown to elicited a significantly higher cytokine response when compared to that from binding to one antigen alone.

Bi-specific, trivalent, and tandem CARs are all known as “OR-gate” CARs, as binding of either CAR to its antigen is sufficient to drive full T cell activation. However, targeting multiple antigens may increase the safety concern especially when treating solid tumors. More specifically, many antigens that are being targeted with CAR T cells also are expressed in normal cells at low levels, raising the risk of on-target off-tumor toxicities. To address such issues, the split CARs, also known as “AND-gate” CARs, were designed. With this approach, T cells were engineered to co-express two different CARs, one containing the CD3ζ signal and the other containing the co-stimulation signal. Recognition of a single antigen in normal cells will not be enough to activate these CAR T cells, however, only when bound to both antigens, which are overexpressed in tumor cells, would the T cells be fully activated (Fig. 2f). While not reported in GBM, Lanitis et al*.* [34] generated CAR T cells that co-express anti-mesothelin scFv-CD3ζ and anti-α-folate receptor (FRα) scFv-CD28 CARs. These AND-gate CAR T cells showed weak cytokine secretion against the target cells expressing only one antigen in vitro*.* When tested in vivo, the mesothelin/FRα-CAR T cells showed potent efficacy against tumors overexpressing both FRα and mesothelin, but not normal tissues expressing mesothelin alone.

The synNotch CAR is another “AND-gate” CAR [35]. The Notch protein is a transmembrane receptor with an intracellular domain containing a transcriptional regulator that is released from the membrane when activated by its ligand. With this mechanism, a synthetic Notch receptor (synNotch) was designed for binding to the first antigen for activation of synNotch system, which then switches-on the transcription of a CAR (synNotch CAR) to recognize and bind to the second antigen on cancer cells (Fig. 2g). Thus, overexpression of both antigens is required to provide the signal needed for CAR T cell killing and activation. While this system has showed functionality in vitro and in in vivo animal models [36, 37], the applications for brain tumors as well as in clinical trials have not been established.

### Genetic modification to improve CAR T cell persistence and safety

Since CAR T-cell therapy showed promising but limited anti-tumor response in GBM clinical trials in 1990s, efforts have been made to improve CAR T-cell efficacy. A strategy is to further engineer T cells to overexpress transgenic proteins such as cytokines and chemokines that are known to stimulate T cell proliferation and persistence. Such CAR T cells are also known as “T cells redirected for universal cytokine-mediated killing”, so-called TRUCKs or the 4th generation CARs [23] (Fig. 1b). Studies have shown that modifying CAR structures to overexpress IL-15 or constitutively active IL-7R (C7R) improved CAR T cell anti-tumor activity and persistence in GBM [38, 39]. A recent study further reported that an “All-in-One” lentiviral TRUCKs which overexpress IL-12 or IL-18 dependent on CAR T cell activation improved the efficacy of GD2-CAR T cells in GBM [40]. While these additional CAR T cell modifications greatly improved CAR T-cell therapy, they pose the risk of uncontrollable T cell proliferation resulting in potential treatment-related toxicities.

Safety and toxicity have always been a concern since CAR T-cell therapy entered the clinical trials. The major reported toxicity is cytokine release syndrome (CRS) as determined by a rapid and sustained cytokine release including IL-2, IL-6, IL-10 and IFNγ following T cell activation and expansion [41–43]. However, great progress has been achieved since tocilizumab, an FDA-approved anti-IL-6 antibody, showed rapid reversal of severe CRS syndromes, which has become the standard management [44].

As mentioned above, split CARs and synNotch CARs are designed to precisely target tumor cells rather than normal tissues, reducing the risk of toxicity. A more specific approach to eliminate unintended adverse effects is to manage CAR T-cell survival by modifying CAR vectors to include an inducible “suicide” gene as a safety switch. For example, Stavrou et al*.* [45] has reported an approach using rapamycin administration to activate the expression of caspase 9 (an apoptotic protease) in CAR T cells, which may drive CAR T cells toward apoptosis when needed. Notably, use of the inducible caspase 9 safety switch has entered clinical trials for treating relapsed or refractory ALL (NCT03016377) and B-cell lymphoma (NCT03696784). While severe toxicity has not been reported in GBM clinical trials, methods are being developed to avoid potential risks. Rituximab is a mono-clonal antibody that targets CD20 cells and leads to the depletion of B lymphocytes. To reduce the potential toxicity of targeting EphA2 in GBM, Yi et al*.* [46] showed that using CD20 as a tag in the CAR vector not only served as a selection marker for determining CAR T-cell transduction efficacy using flow cytometry, but also acted as a safety switch. Upon stimulation with rituximab, the activated CAR T cells can be efficiently eliminated within 24 h of treatment. Other safety switches include EGFR (targeted by cetuximab) [47, 48], and herpes simplex virus thymidine kinase (targeted by ganciclovir) [49].

## CAR T-cell therapeutic targets for GBM

### CAR T cell targets that are in clinical trials against GBM

CAR T cell targets for brain tumors that have been evaluated pre-clinically and in early phase clinical trials are IL13Rα2, EGFRvIII, HER2, EphA2, GD2, B7-H3 and chlorotoxin. Below we discuss each of these targets by reviewing their biology and clinical trial outcomes (Table 1).

#### IL13Rα2

Interleukin-13 receptor alpha 2 (IL13Rα2) overexpression occurs in over 75% of GBM tumors, and is associated with invasive glioma growth and poor prognosis [50]. In normal cells, IL-13 binds to IL13Rα1 monomers with low affinity, forming a heterodimer with IL4Rα, followed by activating STAT6 to induce the pro-apoptosis activity. However, in glioma, IL13Rα2 binds to IL-13 with higher affinity, allowing for sequestration of IL-13 away from IL13Rα1 [51, 52]. The fact that IL13Rα2 is specifically overexpressed in GBM but not normal tissue and with higher affinity binding sites than IL13Rα1 has made it the first CAR T cell target for GBM. Brown et al*.* have reported that administration of IL13Rα2-specific CAR T cells into the post-surgical cavity of GBM patients resulted in a 7.5-month regression period with a median overall survival of 11 months without severe toxicity [50, 53]. Despite the initial positive response in clinical trials, IL13Rα2-specific CAR T-cell therapy is being challenged by tumor recurrence due to antigen loss and limited T cell persistence [50]. Thus, methods aiming at improving IL13Ra2-specific CAR T-cell efficacy have been evaluated [38]. In addition, evaluation of the combination therapy of IL13Rα2-specific CAR T cells with the immune checkpoint inhibitors (ICI), such as nivolumab and ipilimumab, are in clinical trials (NCT04003649).

#### EGFRvIII

EGFR is a well-known receptor tyrosine kinase (RTK) involved in cancer initiation and progression. The EGFRvIII is the most common EGFR mutation that occurs in about 45% of GBM patients. EGFRvIII arises from the deletion of exons 2–7 and has a truncated extracellular domain. This mutation causes constitutive activation of RTK/RAS/AKT signaling which is enhanced in 88% of GBM patients [54]. EGFRvIII is specific to GBM and is not found in normal tissues. EGFRvIII targeting inhibitors failed to show significant clinical efficacy in GBM patients mainly due to signaling bypass, in which inhibition of EGFR vIII pathway leads to activation of other RTKs, providing a good reason for further developing EGFR vIII-CAR T-cell therapy [55]. O’Rourke et al*.* [41] reported the first study in humans of intravenous delivery of a single dose of EGFRvIII-CAR T cells in 10 recurrent GBM patients which showed safety and limited anti-tumor response as well as targeted antigen downregulation. Goff et al. [56] reported a pilot phase I trial with third generation EGFRvIII-CAR T cells administered after lymphodepleting chemotherapy and intravenous interleukin-2 injections. This study did not show significant toxicity but failed to show clinical efficacy. Another design of EGFRvIII-CAR T cell using humanized scFv again proved safe but also failed to show clinical benefit [41, 57]. Currently, there are six EGFRvIII-CAR T cell clinical trials ongoing with two in combination with chemotherapy or ICI (Table 1).

#### HER2

HER2 is a member of the EGFR family, also named as ERBB2. Increased levels of HER2 protein in GBM patients is linked to poor survival [14, 58, 59]. Unlike EGFRvIII, which is unique to GBM, HER2 is overexpressed in many cancer types including breast, ovarian and GBM; it also is expressed in some normal tissues, leading to safety concerns. An early case report with HER2-CAR T cell therapy has documented a metastatic colon cancer patient who experienced respiratory distress within 15 min of HER2-targeting CAR T cell administration and died 5 days after treatment. Serum samples after cell infusion showed marked increases in interferon γ (IFNγ), granulocyte macrophage-colony stimulating factor (GM-CSF), tumor necrosis factor-alpha (TNF-alpha), interleukin-6 and -10 (IL-6 and IL-10), consistent with a cytokine storm [60]. Since then, efforts have been made to overcome these safety concerns [61]. Liu et al*.* [62] showed that lowering the binding affinity between scFv and HER2 may increase the differential binding of CAR T cells to tumor versus normal tissue in preclinical cancer models, providing a good strategy for targeting the antigens that are not specific to solid tumors. A more recent clinical trial (NCT00902044) reported no significant toxicities of a second generation HER2-specific CAR T-cell therapy for sarcoma [63]. Compared with the original 4D5 HER2-CAR which binds to the juxtamembrane region of HER2 protein and caused severe toxicity, this trial used a FRP5-HER2 CAR which recognizes a discontinuous epitope between residues 11–169 of HER2 that is more distant from the cell surface. Thus, the binding site of the epitope also determines the activity of the CARs. As for GBM, investigators at Baylor College of Medicine have conducted a clinical trial (NCT01109095) to evaluate the safety and efficacy of HER2-specific CARs using virus-specific T cells (CAR-VSTs) [59]; previous studies have shown that virus engineered cytotoxic T cells demonstrate better persistence and T cell expansion through appropriate co-stimulatory signaling activation [64]. In this trial, overexpression of FRP5 HER2-CARs in virus-specific T cells not only improved the specific targeting of HER2^+^ tumor cells, but also prolonged the HER2-CAR VST cell persistence and antitumor activity. While this trial has demonstrated treatment safety, the clinical benefit was limited.

#### EphA2

EphA2 protein is overexpressed in gliomas and is associated with malignancy, thus it became a good molecular target in GBM [65, 66]. Preclinical studies demonstrated that a second generation EphA2-CAR T cell induced glioma xenograft tumor regression in vivo [46, 67]. So far one clinical trial was initiated to evaluate the safety and effectiveness of CAR T cell immunotherapy in treating patients with EphA2^+^ malignant glioma but has been withdrawn recently for unknown reason (NCT02575261).

#### GD2

GD2 is a glycosphingolipid expressed at low levels on the surface of healthy cells, but highly expressed on several tumor types including gliomas and is associated with increased tumor proliferation and invasion [68]. Pre-clinical studies have demonstrated a robust antigen-dependent cytokine production and killing of GD2^+^-positive glioma cells in vitro and in vivo using patient-derived orthotopic xenograft models overexpressing GD2 [16, 17]. Currently, two phase I clinical trials (NCT04099797 and NCT03423992) are evaluating the safety and efficacy of GD2-specific CAR T cells in high grade glioma and diffuse intrinsic pontine glioma (DIPG).

#### B7-H3

B7-H3 (CD276) is an immune checkpoint molecule and a member of B7 protein superfamily. B7-H3 binds to the majority of neuroepithelial tumors but not to normal glia or tissues, making it a promising target for therapeutics [69]. Clinical trials for two mAbs targeting B7-H3 (8H9 and MGA271) have been shown to be safe and promising for metastatic CNS neuroblastoma and DIPG in children.[70, 71]. Using a B7-H3 (MGA271) 41BBζ CAR, Majzner et al. [18] have reported a robust anti-tumor activity in multiple solid, liquid, and CNS tumor types. In addition, Nguyen et al. [72] demonstrated that B7-H3-specific (MGA271) CD28ζ CAR-T cells have potent anti-tumor activity in U373 glioma model. To date, two GBM-related clinical trials focused on B7-H3 CAR T-cell therapy (NCT04385173, NCT04077866) are in the recruitment phase.

#### Chlorotoxin

Chlorotoxin (CLTX) is a 36-amino acid peptide first isolated from scorpion venom that specifically binds to GBM but not to normal tissue. Pharmacologically, CLTX binds to and blocks small-conductance chloride channels [73]. Unlike the common CARs that are designed to recognize surface TAAs to kill tumor cells, the CLTX-CAR was designed as a peptide-based CAR to recapitulate the GBM-binding potential of CLTX. With this approach, Wang et al. [19] showed that CLTX-CAR T cells mediate potent anti-GBM activity and efficiently targeted tumors lacking expression of other GBM-associated antigens, resulting in tumor regression in orthotopic xenograft GBM tumor models. Importantly, the CLTX-CAR-T cells exhibited minimal off-target effects, without showing toxicity following systemic or regional delivery into mice. Given the finding that effective targeting by CLTX-CAR T cells requires cell surface expression of matrix metalloproteinase-2 (MMP2), a clinical trial has been initiated for treating MMP2^+^ recurrent GBM (NCT04214392). More importantly, this study also opened a new avenue to repurpose the use of a natural toxin for CAR T cell engineering.

### Other potential CAR T cell targets for GBM

While the current CAR T cell targets continue to be developed and improved, progress also is being made in exploring new targets specific for GBM. Below we review other promising brain TAAs that have been tested pre-clinically.

#### CD70

CD70 is a type II transmembrane protein and a member of the tumor necrosis factor family. CD70 was not detected in normal peripheral and brain tissues but was constitutively overexpressed in isocitrate dehydrogenase (IDH) wild-type primary low-grade gliomas, and GBMs in the mesenchymal subgroup and recurrent tumors. CD70 is associated with poor survival in GBM patients making it a good candidate for CAR T-cell therapy [74, 75]. Several pre-clinical studies with differently designed CD70-specific CAR-T cells have shown robust anti-tumor response against CD70^+^ mouse models [74, 76]. While there is no current clinical trial for testing CD70-specific CAR-T cells in GBM, phase I/II clinical trials are underway to study the safety and efficacy of CD70-specific CAR-T cell therapy in B cell cancers (NCT03125577, NCT04429438) as well as pancreatic, renal, breast and ovarian cancers (NCT02830724).

#### CD133

CD133 is a marker for self-renewing cancer stem cells (CSCs) in solid tumors. CD133^+^ tumor cells, including GBM, are known to be highly resistant to chemo- and radiotherapy. Recently, CD133 has been identified as a potential CAR T cell target for treatment of GBM. While CD133 also is expressed on hematopoietic stem and progenitor cells (HSPC), Vora et al*.* [77] demonstrated that intra-tumoral injections of CD133-specific CAR T cells are effective at eliminating GBM and that this treatment does not cause systemic toxicity in humanized mouse models. In addition, in a phase 1 clinical trial (NTC 02541370) CD133-specific CAR T cells showed feasibility and efficacy against advanced metastatic malignancies with no serious adverse effects, further confirming the therapeutic potential of targeting CD133 [78].

#### MET

MET is the receptor of hepatocyte growth factor (HGF), and a well-known RTK being developed as an anti-cancer target. HGF/MET overexpression frequently occur in GBM patients and is associated with poor prognosis [54, 79, 80]. Using a transgenic mouse model, Qin et al*.* [81] showed that overexpression of HGF and MET may transform neuro stem cells into glioma stem cells (GSCs), leading to GBM initiation. GBM harboring MET amplification or HGF autocrine activation are sensitive to MET inhibitors in preclinical models [82, 83], recent clinical trials further showed that a combination of MET and VEGF inhibitors (ontarzucimab plus bevacizumab vs. placebo plus bevacizumab) significantly improved progression free survival (PFS) and overall survival (OS) in the mesenchymal subtype of recurrent GBM patients with high tumor HGF expression [84]. These results suggest that MET maybe a good target for CAR T-cell therapy in GBM.

### Summary for CAR T cell targets in GBM

Although a large number of CAR T cell targets under development for treating GBM are showing promising preclinical results, limited antitumor response has been observed in clinical trials, largely due to limited T cell persistence and antigen-negative relapses. While technical innovations to improve the CAR T cell expansion, survival, efficacy and safety remain the key next steps to pursue, combination with other therapies also have been comprehensively considered to improve the CAR T cell therapy [85]. Below, we introduce the combination with ICIs as a promising approach to overcome T cell exhaustion which in turn may improve CAR T cell efficacy in clinical trials.

## CAR T cell therapy in combination with immune checkpoint inhibitors

The tumor microenvironment is orchestrated by immunosuppressive cytokines, regulatory modulators and co-inhibitory receptors that regulate the responsiveness of immune cells [86]. While active CAR T cells elicit specific recognition and killing activities against tumor cells, the chronic exposure to tumor cells results in T cell exhaustion mediated by immune checkpoint pathway activation that is initiated by tumor cells, leading to a reduced ability to proliferate, produce cytokines and attack the tumor (Fig. 1c). Exhausted T cells upregulate immune inhibitory receptors, such as programmed death ligand-1 (PD-L1) and cytotoxic T lymphocyte-associated antigen 4 (CTLA-4). Blocking this immune suppressive signaling has led to the development of ICIs such as nivolumab and durvalumab (PD-1/PD-L1 inhibitors) and ipilimumab (CTLA-4 inhibitors). These ICIs are FDA-approved for treating several types of cancer including melanoma, hepatocellular carcinoma and lymphoma and have shown significant clinical results [87]. Since ICIs eliminate T cell exhaustion, combining their use may further enhance CAR T-cell therapy efficacy through extended T cell proliferation and sustained tumor-killing activity. A recent case report has shown that administration of the PD-1/PD-L1 inhibitor pembrolizumab after CD19-specific CAR T-cell therapy in refractory diffuse large B-cell lymphoma induced a clinically significant antitumor response, suggesting that the PD-1 pathway may be critical in determining the response to CAR-modified T-cell immunotherapy [88]. However, despite the promising clinical results in other types of cancer, the use of ICIs in GBM clinical trials remain controversial. Nivolumab, a PD-1/PD-L1 inhibitor, failed to prolong overall survival of patients with recurrent GBM, leading to a discussion of whether ICIs may benefit GBM patients after all [89–91]. Nevertheless, a recent trial with 35 patients with recurrent, surgically-removable GBM showed that patients who receive neoadjuvant PD-1 blockade, with continued adjuvant therapy following surgery had significantly better overall survival compared to those who received adjuvant or post-surgical PD-1 blockade alone [92]. Another study also suggests that combination of local chemotherapy with PD-1 blockade enhanced antigen-specific T effector cell expansion and improves survival in GBM models [93]. These studies suggest that anti-PD-1 blockade alone may not directly benefit GBM patients but may improve the efficacy of other therapeutics when used in combination. Currently, clinical trials are on-going to evaluate the therapeutic efficacy of EGFRvIII-CAR (NCT03726515) and IL13Rα2-CAR (NCT04003649) T-cell therapy in combination with ICIs such as pembrolizumab or nivolumab in recurrent or refractory GBM patients (Table 1). We anticipate that anti-PD1 blockage may improve the CAR T-cell efficacy for treating GBM patients.

## Future perspectives

Over the past decades, we have learned key principles to manage CAR T-cell efficacy: the specificity of the targeted antigen, the sufficient TCR activation by the specific antigen binding domain, the level of T cell activation and longevity, and the hostile micro-environment for T cells to penetrate, which also serves a source of immunosuppressive factors [94]. While many types of CAR T cells are now in clinical trials (Table 1), further optimization with advanced approaches are expected to improve their overall clinical efficacy.

Since genetic modification of CAR vectors is showing promising results in improving CAR T-cell efficacy, gene editing of the T cell is now a new strategy to further improve CAR T cells. Recently, the CRISPR/Cas9 mediated gene editing system has been applied to delete or mutate checkpoint-associated genes in CAR T cells, and has shown improvement in T cell persistence and survival [95–97]. Using CRISPR/Cas 9-mediated deletion, Ren et al*.* [95] generated CD19-CAR T cells simultaneously deficient in TCR, HLA class I molecule and PD1, which showed more potent antitumor activities than non-edited CAR T cells when tested in xenograft mouse models. Notably, the TCR and HLA class I double-deficient T cells further reduced MHC restriction and eliminated alloreactivity and potential graft-versus-host disease (GVHD), thus making the strategy particularly useful for treating different patients. A different study using CRISPR/Cas9-mediated deletion of TCR α subunit constant (TRAC) region, beta-2 microglobulin (B2M) and PD-1 also showed profound anti-tumor activity in vivo [97]. Recently, Li et al*.* [98] investigated the approaches to improve CAR T transduction efficacy and CRISPR/Cas9 mediated PD-1 deletion using a 2-in-1 lentivirus vector and found that blocking anti-viral signaling in human primary T cells enhanced lentiviral-mediated transduction efficacy of a combinatory CAR/CRISPR vector for targeting HER2 and inhibiting PD-1 at the same time. Most importantly, the first phase 1 clinical trial using CRISPR/Cas9 for multiplex gene editing on T cells (NCT03399448) has demonstrated safety and feasibility in three patients with refractory cancer [99]. Given that the CRISPR/Cas9 approach has been widely applied to functional gene screening and validation, it also opens a new avenue to discover and target novel modulators of immune suppression. Through CRISPR/Cas9-mutagenesis screening, Wei et al. [100] have identified Regnase-1 as a major negative regulator of anti-tumor responses in CD8^+^ T cells, and that CD19-CAR T cells with deleted Regnase-1 showed greater longevity and a more vigorous anti-tumor response in B16 ovarian and B6 melanoma mouse models. We anticipate that future GBM-specific studies also will explore these novel ideas and approaches, which hopefully will further enhance CAR T-cell proliferation, longevity and long-term anti-tumor activity.

## Conclusion

Brain tumors are considered one of the “most-difficult-to- treat” solid tumors. With the most recent advances in immunotherapy, the CAR T-cell therapy has become a revolutionary approach for treating hematological malignancies and it has great potential for brain tumors. This review intends to introduce interpretations of the most relevant papers addressing the use of CAR T cells for the treatment of GBM. We discussed the CAR designs and optimization, the major CAR T cell targets in clinical trials, as well as the strategies developed to improve CAR-T cell efficacy in the context of GBM. We anticipate that future clinical trial designs will not only focus on efficacy and safety, but also on the mechanisms involved in the immune response and resistance, and that the next generation of CAR T cell therapy will become an effective and safe therapeutics for treating malignant GBM.

## Data Availability

All data are available in the manuscript or upon request to the authors.

## Abbreviations

ALL: Acute lymphoblastic leukemia
ACT: Adoptive cell transfer
BBB: Blood brain barrier
B2M: Beta-2 microglobulin
CSC: Cancer stem cells
CAR: Chimeric antigen receptors
CRISPR: Clustered regularly interspaced short palindromic repeats
Cas9: CRISPR-associated protein 9
CRS: Cytokine release syndrome
CTLA-4: Cytotoxic T lymphocyte-associated antigen 4
DIPG: Diffuse intrinsic pontine glioma
EGFRvIII: Epidermal growth factor receptor variant III
EphA2: Erythropoietin-producing hepatocellular carcinoma A2
GBM: Glioblastoma multiforme
GVHD: Graft-versus-host disease
GD2: Ganglioside 2
Frα: Folate receptor α
HGF: Hepatocyte growth factor
HER2: Human epidermal growth factor 2
IL13Rα2: Interleukin-13 receptor alpha 2
HSPC: Hematopoietic stem and progenitor cells
IDH: Isocitrate dehydrogenase
GM-CSF: Granulocyte macrophage-colony stimulating factor
TNF-alpha: Tumor necrosis factor-alpha
IL: Interleukin
IFNγ: Interferon γ
MHC: Major histocompatibility complex
MMP2: Matrix metalloproteinase-2
OS: Overall survival
PD-1: Programmed death-1
PD-L1: Programmed death ligand-1
PFS: Progression free survival
scFv: Single chain variable domain of monoclonal antibodies
SUPRA CAR: Split, universal and programmable CAR
TAA: Tumor associated antigen
TCR: T cell receptors
TRAC: TCRα subunit constant
TRUCKs: T cells redirected for universal cytokine-mediated killing
uCAR: Universal CAR
